# Calcium hydroxide nanoparticles induce cell death, genomic instability, oxidative stress and apoptotic gene dysregulation on human HepG2 cells

**DOI:** 10.1038/s41598-025-86401-4

**Published:** 2025-01-23

**Authors:** Hanan R. H. Mohamed, Esraa H. Ibrahim, Shahd E. E. Shaheen, Nesma O. E. Hussein, Ayman Diab, Gehan Safwat

**Affiliations:** 1 Zoology Department Faculty of Science, Cairo University, Giza, Egypt; 2 Faculty of Biotechnology, October University for Modern Sciences and Arts, 6th of October, Egypt

**Keywords:** Calcium hydroxide nanoparticles, DNA breaks, ROS generation, Apoptosis induction, HSF and HepG2 cells, Genetics, Molecular biology, Health care

## Abstract

Calcium hydroxide nanoparticles (Ca(OH)_2_NPs) possess potent antimicrobial activities and unique physical and chemical properties, making them valuable across various fields. However, limited information exists regarding their effects on genomic DNA integrity and their potential to induce apoptosis in normal and cancerous human cell lines. This study thus aimed to evaluate the impact of Ca(OH)_2_NPs on cell viability, genomic DNA integrity, and oxidative stress induction in human normal skin fibroblasts (HSF) and cancerous hepatic (HepG2) cells. Cell viability and genomic DNA stability were assessed using the Sulforhodamine B (SRB) assay and alkaline comet assay, respectively. Reactive oxygen species (ROS) levels were measured using 2,7-dichlorofluorescein diacetate, while the expression level of apoptosis-related genes (p53, Bax, and Bcl-2) were quantified using real-time PCR (qRT-PCR). The SRB cytotoxicity assay revealed that a 48-hour exposure to Ca(OH)_2_NPs caused concentration-dependent cell death and proliferation inhibition in both HSF and HepG2 cells, with IC50 values of 271.93 µg/mL for HSF and 291.8 µg/mL for HepG2 cells. Treatment with the IC50 concentration of Ca(OH)_2_NPs selectively induced significant DNA damage, excessive ROS generation, and marked dysregulation of apoptotic (p53 and Bax) and anti-apoptotic (Bcl-2) gene expression in HepG2 cells, triggering apoptosis. In contrast, exposure of HSF cells to the IC50 concentration of Ca(OH)_2_NPs caused no significant changes in genomic DNA integrity, ROS generation, or apoptotic gene expression. These findings indicate that Ca(OH)_2_NPs exhibit concentration-dependent cytotoxicity in both normal HSF and cancerous HepG2 cells. However, exposure to the IC50 concentration was non-genotoxic to normal HSF cells while selectively inducing genotoxicity and apoptosis in HepG2 cancer cells through DNA breaks and ROS-mediated mechanisms. Further studies are required to explore the biological and toxicological properties and therapeutic potential of Ca(OH)_2_NPs in hepatic cancer treatment.

## Introduction

Calcium hydroxide nanoparticles (Ca(OH)_2_NPs) have garnered significant interest across diverse fields due to their distinctive physicochemical properties. These include high reactivity, a large surface area-to-volume ratio, enhanced cell membrane penetration, and superior antimicrobial activity compared to their bulk counterparts. In medicine, Ca(OH)_2_NPs are utilized in dental materials and wound healing applications. Similarly, in the construction industry, they play a role in concrete production and wastewater treatment^1–3^.

As the use of Ca(OH)_2_NPs expands across various industries and medical fields, the potential for human exposure to these nanoparticles is increasing. Common exposure routes include inhalation, ingestion, and skin contact, contributing to their presence in everyday products and environments^4,5^. This heightened exposure has raised concerns regarding their potential health impacts, prompting extensive research into the safety and toxicity of Ca(OH)_2_NPs^6,7^.

The toxicity of Ca(OH)_2_NPs remains a subject of ongoing research and debate. While these nanoparticles offer significant potential across various applications, their possible health risks warrant careful consideration. Studies have shown that prolonged exposure to high concentrations of Ca(OH)_2_NPs can lead to respiratory issues, skin irritation, oxidative stress, and other health concerns^5,8^. However, research by de Souza and colleagues highlights the safety of Ca(OH)_2_NPs in human dental pulp mesenchymal cells, demonstrating that exposure did not compromise cell viability and even reduced the production of reactive oxygen species (ROS) and nitric oxide^9^.

The genotoxic effects of Ca(OH)_2_NPs have attracted considerable attention in recent studies. In vivo studies have revealed their genotoxicity, showing that exposure to Ca(OH)_2_NPs can cause genomic and mitochondrial DNA damage, excessive ROS generation, and alterations in inflammatory and apoptotic gene expression in tissues such as bone marrow, liver, brain, stomach, and kidneys in mice^3,4,6,10^.

Understanding the cytotoxicity and genotoxicity of Ca(OH)_2_NPs across a broader range of cell lines and experimental systems is crucial for evaluating their safety, developing effective risk management strategies, and exploring their therapeutic potential. However, existing data are limited, with only a few studies examining the cytotoxic and genotoxic effects of Ca(OH)_2_NPs on different human normal and cancer cell lines.

To address this gap, the present study was conducted to investigate the impact of Ca(OH)_2_NPs exposure on cell viability, genomic DNA integrity, and ROS generation in human normal skin fibroblast (HSF) and hepatocellular carcinoma (HepG2) cells. Cell viability was assessed using the Sulforhodamine B (SRB) cytotoxicity assay, while genomic DNA integrity and ROS generation level was evaluated using the alkaline Comet assay and 2’,7’-dichlorodihydrofluorescein diacetate (DCFDA) dye, respectively. The expression level of apoptosis related genes was also measured using quantitative real time PCR (qRT-PCR).

## Materials and methods

### Chemicals

White powders of Ca(OH)_2_NPs were purchased from Nano Tech company Egypt with size less than 100 nm. Prior treatment, Ca(OH)_2_NPs were suspended and ultra-sonicated in deionized distilled water to prepare the tested concentrations. All the remaining used chemicals and reagents in the conducted experiments were of high analytical and molecular biology grade.

### Characterization of ca(OH)_2_NPs

The characterization of Ca(OH)_2_NPs was carried out to ensure their purity and evaluate their physicochemical properties. X-ray diffraction (XRD) analysis was performed using a charge-coupled device diffractometer (XPERT-PRO, PANalytical, Netherlands) to confirm the purity of the purchased nanoparticles. The zeta potential and particle size distribution were analyzed using a Malvern Zetasizer Nano Series (Malvern Instruments, Westborough, MA). Additionally, the morphology and average size of the Ca(OH)_2_NPs were determined through transmission electron microscopy (TEM) imaging.

### Cell lines

Normal Human Skin Fibroblast (HSF) and hepatocellular carcinoma (HepG2) cells were supplied from Nawah Scientific Company (Mokatam Cairo Egypt) and were maintained in Dulbecco’s Modified Eagle Medium (DMEM) media supplemented with 100 mg/mL of streptomycin, 100 units/mL of penicillin and 10% of heat-inactivated fetal bovine serum in humidified, 5% (v/v) CO2 atmosphere at 37 °C.

### Cell viability

The Sulforhodamine B (SRB) assay was performed to evaluate the viability of normal HSF and HepG2 cells following 48 h of treatment with Ca(OH)_2_NPs^11^. A 100 µL suspension containing 5 × 10^3^ cells was seeded into 96-well plates and incubated for 24 h in complete media. After incubation, the cells were treated with 100 µL of media containing Ca(OH)_2_NPs at different concentrations (0.1, 1, 10, 100, and 1000 µg/ml). After 48 h of exposure to the nanoparticles, the cells were fixed by replacing the media with 150 µL of 10% trichloroacetic acid (TCA) and incubating at 4 °C for 1 h. The TCA solution was then removed, and the cells were washed five times with distilled water. Next, 70 µL of 0.4% (w/v) SRB solution was added to each well, and the plates were incubated in the dark at room temperature for 10 min. Unbound dye was removed by washing the plates three times with 1% acetic acid, and the plates were allowed to air-dry overnight. Finally, 150 µL of 10 mM TRIS solution was added to dissolve the protein-bound SRB stain, and the absorbance was measured at 540 nm using an Infinite F50 microplate reader (TECAN, Switzerland).

### Treatment schedule

Normal HSF and cancerous HepG2 cells were cultured separately under the proper conditions and divided into control and treated cells. Control cells were exposed to an equivalent volume of the vehicle (DMSO; final concentration, ≤ 0.1%), while treated HSF and cancer HepG2 cells were exposed to Ca(OH)_2_NPs at the IC50 concentration determined using SRB assay. After 48 h of treatment, both control and treated cells were harvested by trypsinization and centrifugation. Each treatment was done in triplicate, and cells were washed twice with ice-cold PBS and stored at -80 C° for different molecular studies.

### Detection of genomic DNA stability

The impact of Ca(OH)_2_NPs on the integrity of genomic DNA was assessed in the treated and untreated HSF and cancer HepG2 cells using Alkaline Comet assay^12,13^. A 15 µl of cell suspension was mixed with 60 µl of low melting agarose, mixed well and then spread on slides pre-coated with normal melting agarose. After gel hardening, all slides were placed in a lysis buffer with PH of 10 and freshly added triton X-100 and Dimethyl sulfoxide for 24 h in dark. After lysis, slides were washed with distilled water and incubated in an alkaline electrophoresis buffer with PH higher than 12 to unwind double stranded DNA. Then electrophoresis was run for 30 min with current 300 mAmp and 35 Volts. Subsequent to electrophoresis, pH neutralization was done using Tris buffer to reanneal single stranded DNA. After that, DNA was fixed with absolute ethanol for permanent preservation. Finally, slides were stained by ethidium bromide and photography was done using epi-fluorescent microscope at magnification 200 × and fifty comet nuclei were analyzed using Comet Score TM software and DNA damage indicating parameters (tail length, %DNA in tail and tail moment) were measured for each sample.

Tail Length: The distance the DNA fragments migrate from the nucleus. A longer tail indicates more DNA damage.

% DNA in tail: The percentage of total DNA that is found in the tail. A higher percentage indicates more DNA fragmentation.

Tail Moment: A measure that combines the tail length and the percentage of DNA in the tail. It provides a quantitative assessment of DNA damage, with a higher tail moment indicating greater damage.

### Studying the generation level of intracellular ROS

The effect of Ca(OH)_2_NPs on the generation of reactive oxygen species (ROS) within HSF and cancer HepG2 cells was also screened using 2,7-dichlorofluorescein diacetate dye that enters cells passively and reacts with ROS forming the highly fluorescent dichlorofluorescein product^14^. A cell suspension was mixed with 2,7-dichlorofluorescein diacetate dye and Aincubated in dark for 30 min. After incubation this mixture was spread on a clean slide and the emitted fluorescent light was validated using epi-fluorescent at 200 × magnification.

### Measurement of apoptosis related gene expression

Influence of Ca(OH)_2_NPs on the mRNA expression level of p53, Bax and Bcl2 genes in the untreated and treated HSF and cancer HepG2 cells was studied using Quantitative Real Time Polymerase Chain Reaction (qRTPCR). The GeneJET RNA Purification Kit (Thermo scientific, USA) (Thermo scientific, USA) was first used to extract the total cellular RNA that was converted subsequently into complementary DNA (cDNA) using the Revert Aid First Strand cDNA Synthesis Kit (Thermo scientific, USA). The obtained cDNA of p53, Bax and Bcl2 genes were finally amplified using SYBER Green master mix and the primers sequence shown in Table 1^15,16^ by the 7500 Fast system (Applied Biosystem 7500, Clinilab, Egypt). The mRNA expression level of the studied apoptotic and anti-apoptotic genes was then determined using the comparative Ct (DDCt) method after standardization using the housekeeping GAPDH gene expression. The final results were expressed as mean ± S.D.

**Table 1:** List of primer sequences used in qRT-PCR.

Gene	Strand	Primer’s sequences
GAPDH	Forward	5’-GAAGGTGAAGGTCGGAGTCA-3’
	Reverse	5’-GAAGATGGTGATGGGATTTC-3’
BAX	Forward	5’-CCGCCGTGGACACAGAC-3’
	Reverse	5’-CAGAAAACATGTCAGCTGCCA-3’
BCL-2	Forward	5’-TCCGATCAGGAAGGCTAGAGT-3’
	Reverse	5’-TCGGTCTCCTAAAAGCAGGC-3’
P53	Forward	5’-CAGCCAAGTCTGTGACTTGCACGTAC-3’
	Reverse	5’-CTATGTCGAAAAGTGTTTCTGTCATC-3’

### Statistical analysis

The obtained results were displayed as mean ± Standard Deviation (S.D) and were analyzed using the Statistical Package for the Social Sciences (SPSS) (version 20) at the significance level *p* < 0.05. One Way Analysis of Variance (ANOVA) followed by Duncan’s test was done to compare the control HSF cells to the treated HSF cells, control HepG2 cells and treated HepG2 cells.

## Results

### Characterization of ca(OH)_2_NPs

**Fig. 1:**
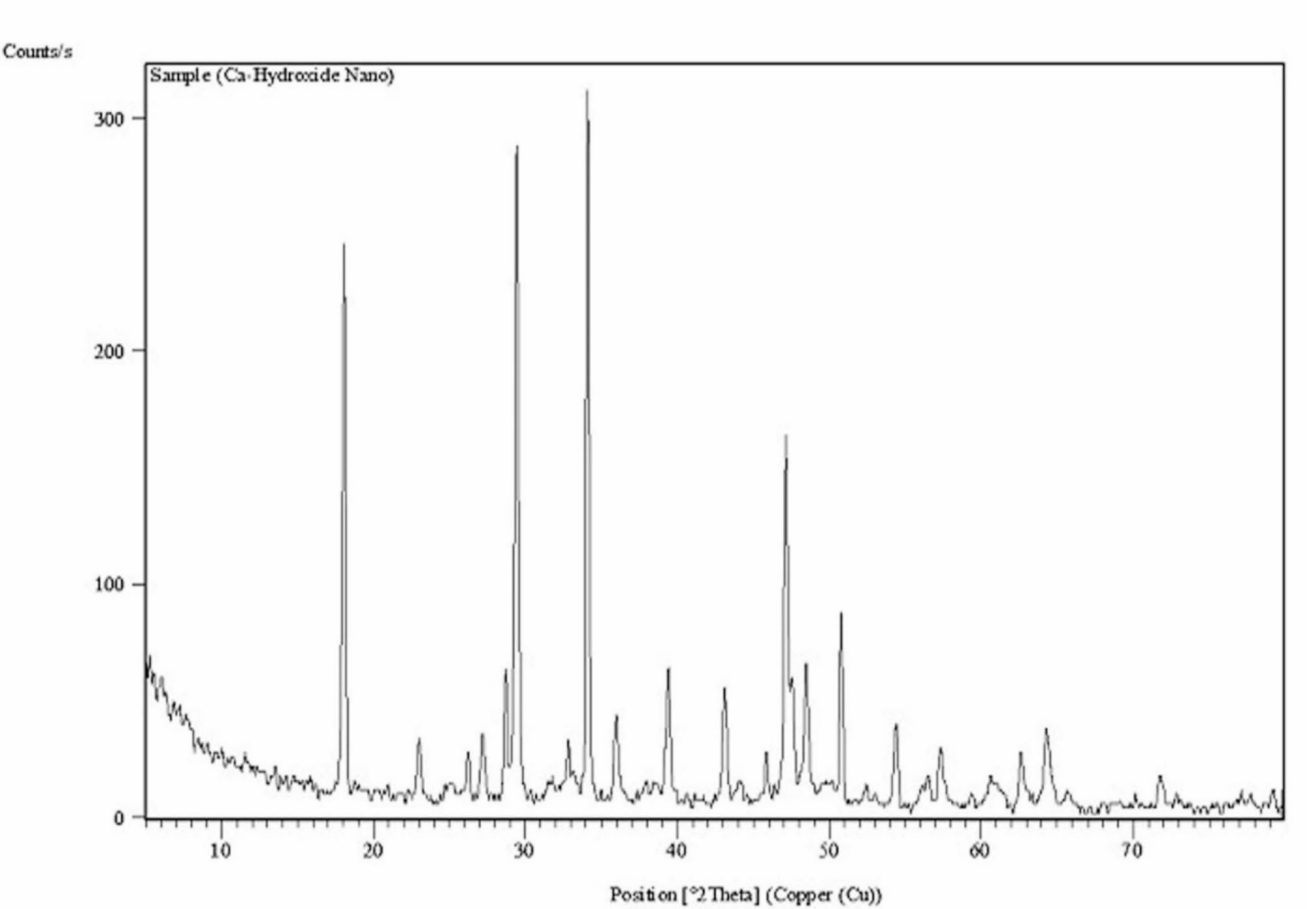
X-ray diffraction (XRD) pattern of Ca(OH)2-NPs.

**Fig. 2:**
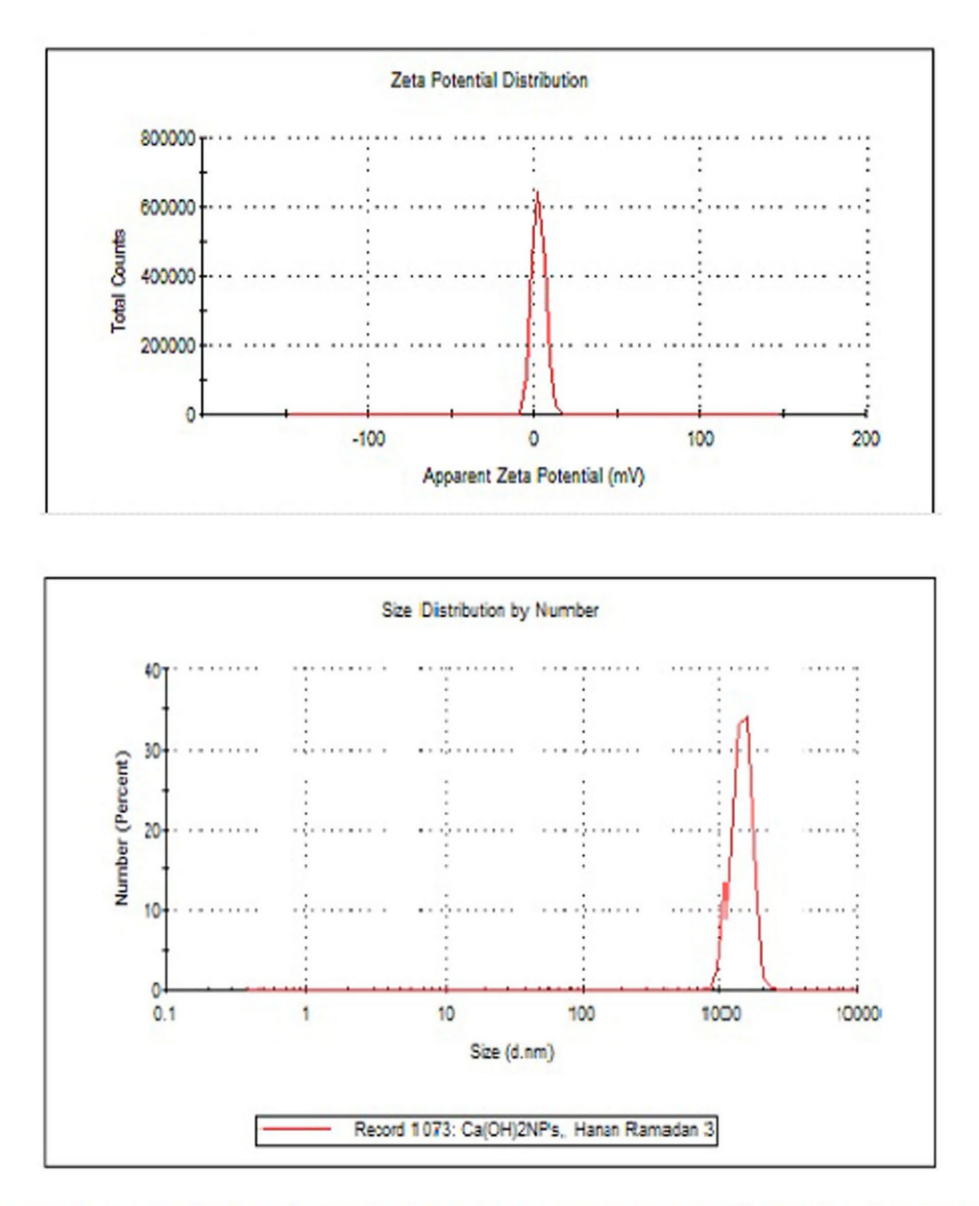
Zeta potential and particle size distribution analysis of Ca(OH)2-NPs.

**Fig. 3:**
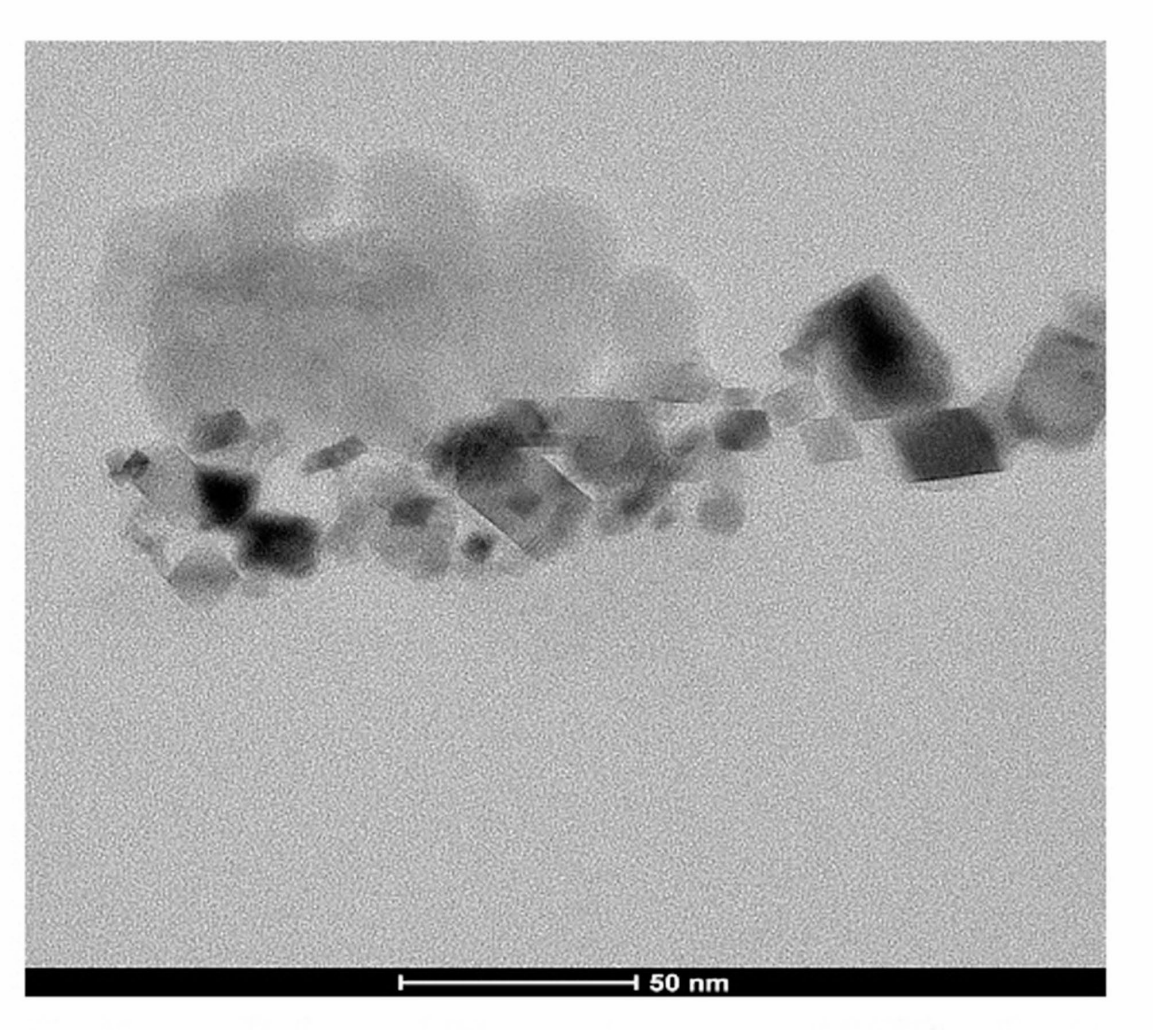
Transmission electron microscope (TEM) imaging of Ca(OH)2-NPs.

Characterization of Ca(OH)_2_NPs using XRD Analysis proved the purity of the purchased nanoparticles by the appearance of distinctive bands for Ca(OH)_2_NPs at the diffraction angles 18º, 29º and 34º (Fig. 1). Analysis of Zeta potential and particles size distribution revealed high aggregation and large hydrodynamic size of the suspended Ca(OH)_2_NPs as manifested from the reported Zeta potential value of 2.45 mV and a polydispersity index (PdI) value of 0.416 for Ca(OH)_2_NPs (Fig. 2). Moreover, imaging of the ultra-sonicated Ca(OH)_2_NPs suspension using TEM showed Ca(OH)_2_NPs have cubic and spherical shape and are well dispersed with an average particle size of 12.4 ± 2.4 nm as seen in Fig. 3.

### Ca(OH)_2_NPs induce death of normal HSF and cancerous HepG2 cells

**Fig. 4:**
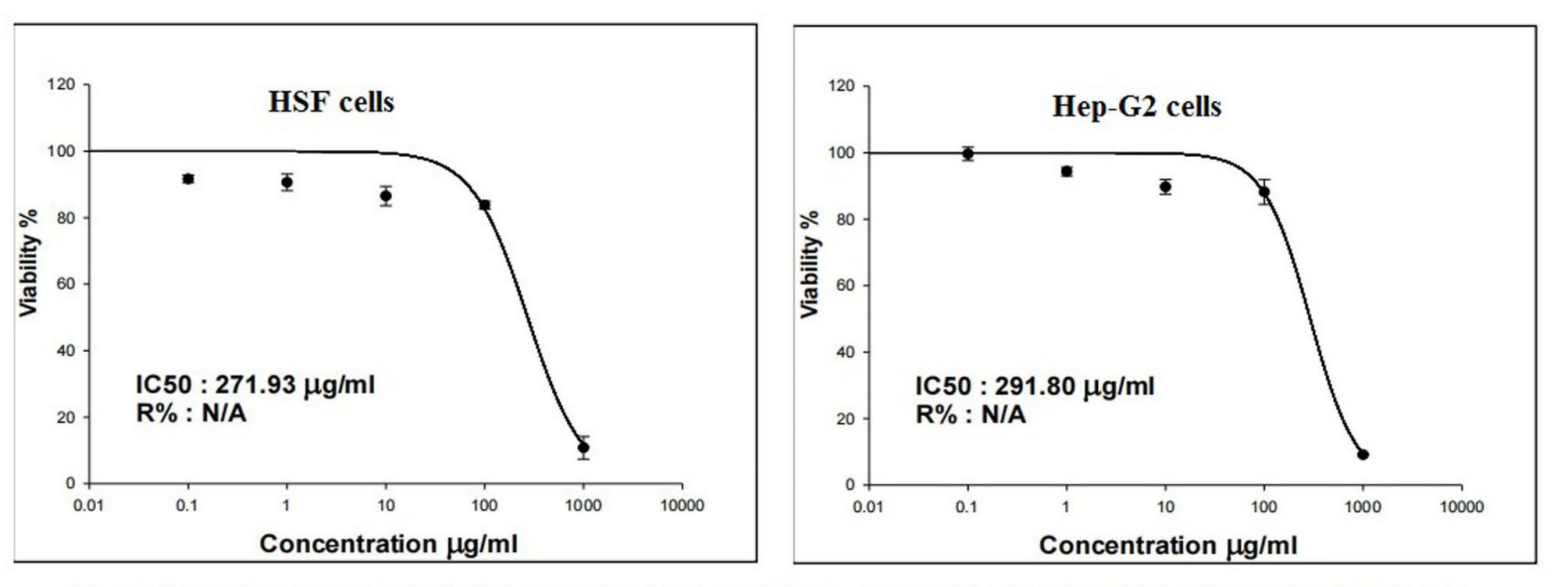
Viability of normal HSF and hepatocellular carcinoma HepG2 cells after 48 h of treatment with five concentrations of Ca(OH)2-NPs.

As seen in Fig. 4 treatment with five different concentrations of Ca(OH)_2_NPs (0.1, 1, 10, 100–1000 µg/ml) caused a marked reduction in the viability of HSF and HepG2 cells only at their highest concentration (1000 µg/ml). The IC50 value of Ca(OH)_2_NPs was equal 271.93 µg/ml for normal HSF cells and 291.80 µg/ml for hepatic cancer HepG2 cells (Fig. 4).

### Ca(OH)_2_NPs selectively induced DNA damage in HepG2 cancerous cells

**Fig. 5:**
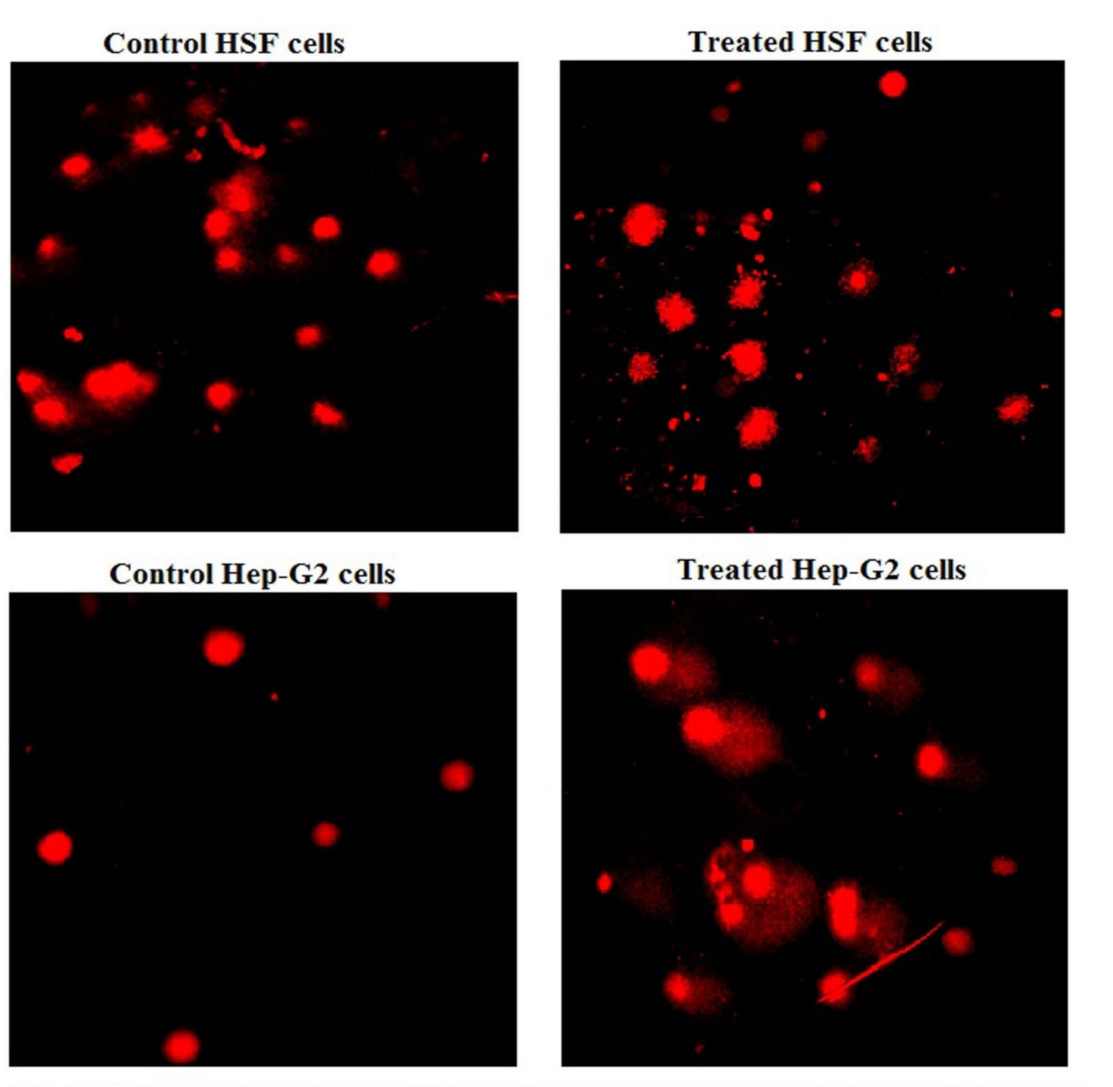
Examples for the scored Comet nuclei with intact and damaged genomic DNA in the control and treated human cells with Ca(OH)2-NPs for 48 h.

The results presented results for alkaline Comet assay in Table 2; Fig. 5 revealed that exposure of HepG2 cells to the IC50 concentration (291.80 µg/ml) of Ca(OH)_2_NPs highly disrupted the integrity of genomic DNA in cancerous HepG2 cells as appeared from the statistical significant elevations (*p* < 0.05) in the measured DNA damage parameters: tail length, %DNA in tail and tail moment seen 48 h after HepG2 cells treatment with Ca(OH)_2_NPs compared to their values in untreated HepG2 cells (Table 2). In contrast, normal HSF cells treatment with IC50 concentration (271.93 µg/ml) of Ca(OH)_2_NPs did not affect the genomic DNA integrity as the measured tail length, and tail moment were non-significantly changed, even %DNA in tail was markedly decreased in the HSF cells treated with Ca(OH)_2_NPs compared to the measured values of untreated HSF cells (Table 2).

**Table 2:** Level of DNA damage induction in normal HSF and cancerous hepatic HepG2 cells treated with IC50 of ca(OH)_2_NPs for 48 h.

Cells	Ca(OH)_2_NPs concentration	Tail length (px)	%DNA in tail	Tail moment
Control HSF cells	0.00	3.45 ± 0.25^a^	15.57 ± 1.88^a^	0.67 ± 0.09^a^
Treated HSF cells	271.93 µg/ml	3.93 ± 0.66^a^	13.97 ± 0.95^a^	0.58 ± 0.22^a^
Control hepatic HepG2 cells	0.00	3.35 ± 0.42^a^	11.09 ± 1.71^a^	0.38 ± 0.03^a^
Treated hepatic HepG2 cells	291.80 µg/ml	10.43 ± 0.36^b^	29.69 ± 2.46^b^	3.24 ± 0.48^b^

Results are expressed as mean ± SD.

Results were analyzed using one-way analysis of variance followed by Duncan’s test to test the similarity between the control and treated HSF and hepatic HepG2 cells.

Means with different letters indicates statistical significant difference at *p* < 0.05 between the compared cells in the same column.

### Ca(OH)_2_NPs selectively dysregulated the mRNA expression level of p53, Bax and Bcl2 genes in HepG2 cancerous cells

The interpretation of qRTPCR results manifested the dysregulation of apoptotic and anti-apoptotic genes expression in the HepG2 cancerous cells treated with Ca(OH)_2_NPs at an IC50 concentration (291.80 µg/ml) for 48 h through the remarkable elevations in the expression level of apoptotic p53 and Bax genes and a noticeable decrease in the anti-apoptotic Bcl2 gene expression observed in the HepG2 cells treated with Ca(OH)_2_NPs compared to their expression level in the untreated HepG2 cells (Table 3). On the other hand, non-remarkable variations were seen in the expression level of p53, Bax and Bcl2 genes 48 h after HSF cells treatment with IC50 concentration (271.93 µg/ml) of Ca(OH)_2_NPs compared to the untreated HSF cells expression level (Table 3).

**Table 3:** Fold change in the expression level of p53, BAX and BCL2 genes in normal HSF and cancerous hepatic HepG2 cells treated with IC50 of ca(OH)_2_NPs for 48 h.

Cells	Ca(OH)_2_NPs concentration	p53 gene	BAX gene	BCL2 gene
Control HSF cells	0.00	1.00 ± 0.00^a^	1.00 ± 0.00^a^	1.00 ± 0.00^a^
Treated HSF cells	271.93 µg/ml	0.99 ± 0.09^a^	1.03 ± 0.04^a^	0.99 ± 0.07^a^
Control hepatic HepG2 cells	0.00	0.13 ± 0.02^b^	0.22 ± 0.03^b^	2.14 ± 0.17^b^
Treated hepatic HepG2 cells	291.80 µg/ml	2.44 ± 0.19^c^	5.33 ± 0.40^c^	0.71 ± 0.03^c^

Results are expressed as mean ± SD.

Results were analyzed using one-way analysis of variance followed by Duncan’s test to test the similarity between the control and treated HSF and hepatic HepG2 cells.

Means with different letters indicates statistical significant difference at *p* < 0.05 between the compared cells in the same column.

### Ca(OH)_2_NPs selectively over-generated ROS within HepG2 cancerous cells

**Fig. 6:**
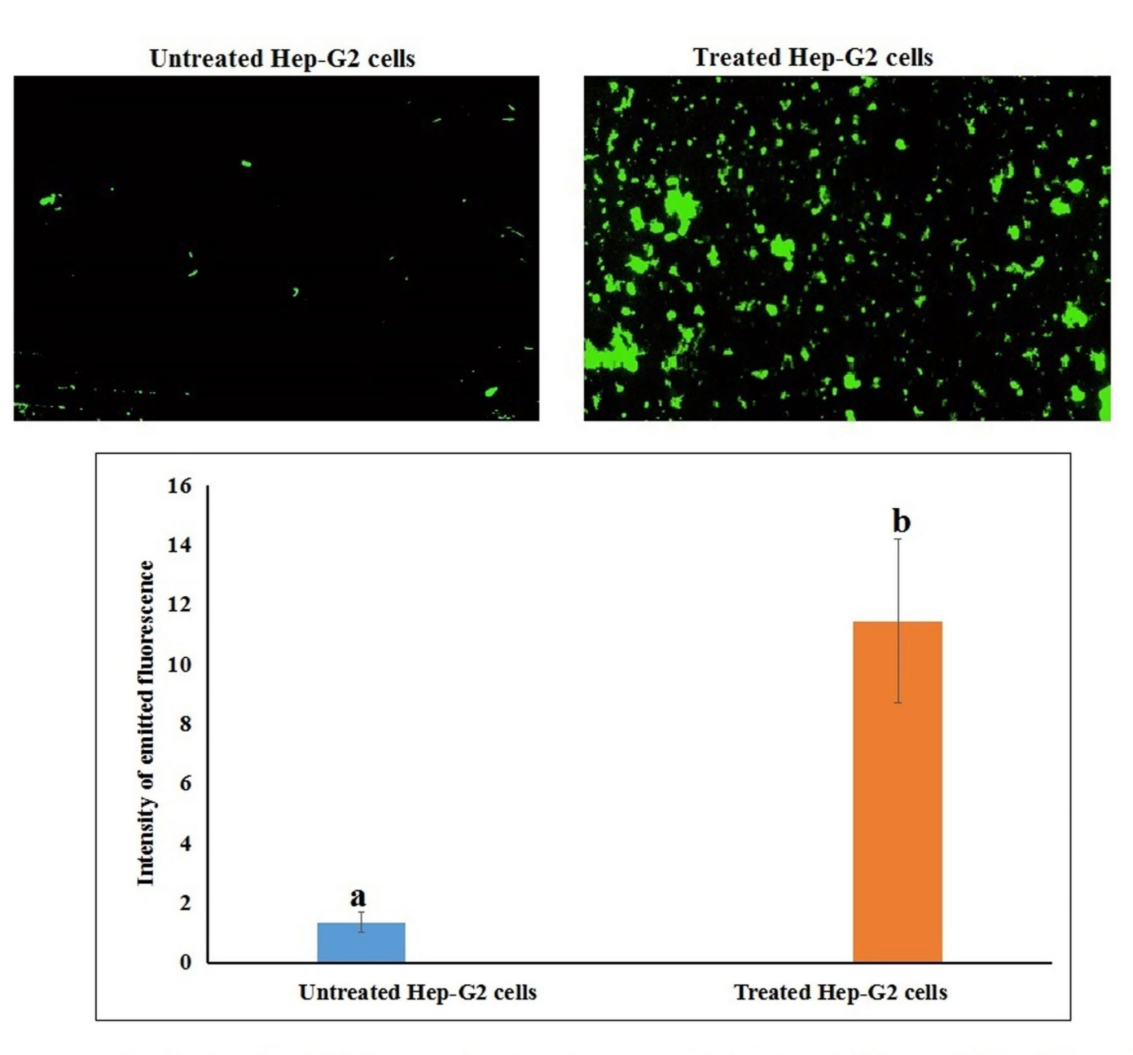
Level of ROS generation within the untreated control and treated human HepG2 cells with Ca(OH)2-NPs for 48 h.

**Fig. 7:**
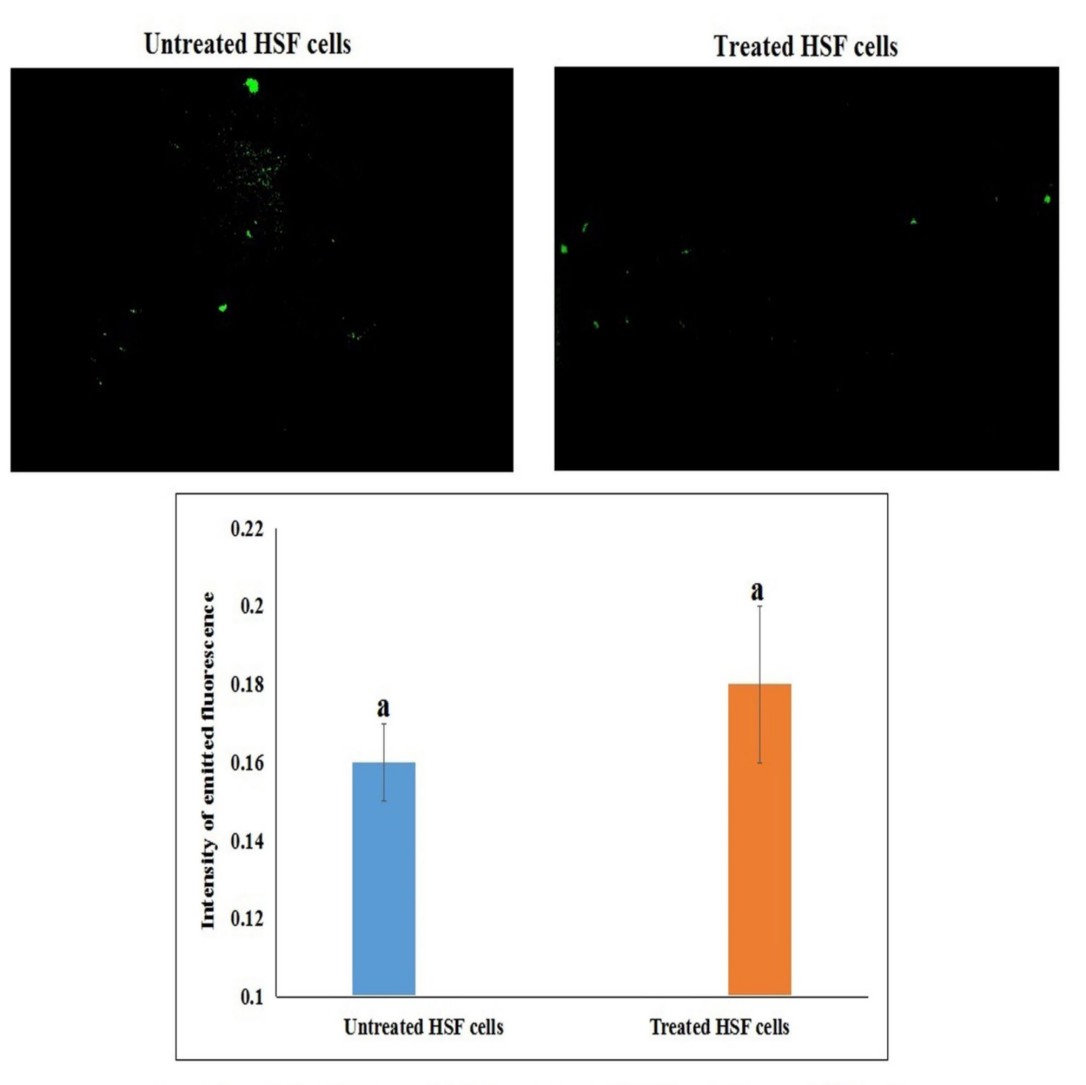
Level of ROS generation within the untreated control and treated human HSF cells with Ca(OH)2-NPs for 48 h.

As displayed in Fig. 6, treatment of HepG2 cancerous cells with the IC50 concentration (291.80 µg/ml) of Ca(OH)_2_NPs for 48 h caused remarkable overproduction of ROS within as shown from the noticeable increase in the intensity of fluorescent light emitted from the treated HepG2 cells compared to that emitted from untreated HepG2 cells (Fig. 6). However, no marked variations were observed in the generation level of ROS after 48 h of HSF treatment with the IC50 concentration (271.93 µg/ml) of Ca(OH)_2_NPs as depicted in Fig. 7 by the non-remarkable changes in the observed in the intensity of fluorescent light emitted from the treated HSF cells compared to that emitted from untreated HSF cells.

## Discussion

The recent discovery of the potent antimicrobial properties of Ca(OH)_2_NPs has increased their uses in biomedicine, environmental science, food industry and agriculture^8^. However, However, there is limited information regarding the impact of Ca(OH)_2_NPs on cell viability and genomic DNA integrity in both normal and cancerous human cells. Therefore, this study was conducted to assess the effect of Ca(OH)_2_NP exposure on cell viability, genomic stability, and ROS generation in normal human HSF cells and cancerous HepG2 cells.

Normal HSF cells are widely used as a model for non-cancerous human tissue in toxicity studies due to their availability, robustness, and ability to provide insights into the general cytotoxic effects of nanoparticles on healthy cells. Similarly, HepG2 cells, a well-established human hepatocellular carcinoma cell line, are extensively employed in cancer research to evaluate anticancer effects, drug delivery systems, and nanoparticle efficacy. Using HSF and HepG2 cells together in this study aligns with previous research^17,18^ and facilitates a comparative assessment of the cytotoxicity of Ca(OH)_2_NPs on normal versus cancerous cells.

The discrepancy between particle size distribution measured by Dynamic Light Scattering (DLS) and Transmission Electron Microscopy (TEM) arises from differences in their principles and contexts. DLS measures the hydrodynamic diameter, which accounts for the particle core as well as any surrounding solvent layer, surface-bound molecules, or adsorbed ions, leading to larger size estimates. In contrast, TEM provides a direct measurement of the core size by imaging the physical dimensions of particles under a microscope. Additionally, DLS is more sensitive to particle aggregates because it detects the collective scattering of light, meaning even minor aggregation can skew the size distribution toward larger values. On the other hand, TEM typically captures particles as individuals due to sample preparation (e.g., drying on a grid), making aggregates less likely to appear unless specifically observed^19,20^.

The cytotoxicity of Ca(OH)_2_NPs was manifested in this study through the marked concentration-dependent proliferation inhibition and reduced viability of normal HSF cells and cancerous HepG2 cells noticed 48 h after Ca(OH)_2_NPs treatment with IC50 values of 271.93 µg/ml for HSF cells and 291.80 µg/ml for HepG2 cells. These results supported the recent study by Prasetyo and his colleagues^21^ that demonstrated the cytotoxicity of Ca(OH)_2_NPs on human umbilical cord mesenchymal stem cells.

Regarding genotoxicity, although Ca(OH)_2_NPs were not genotoxic to normal HSF cells, they were highly genotoxic to HepG2 cancer cells as demonstrated by marked increases in the measured parameters of DNA damage: tail length, %DNA in tail and tail moment observed in Ca(OH)_2_NPs treated HepG2 cells in contrast to the non-significant changes seen in tail length, %DNA in tail and tail moment after HSF cells exposure to Ca(OH)_2_NPs. Alkaline Comet assay is a highly sensitive cyto-molecular genetic technique for detecting DNA damage. It sensitively detects both single- and double-stranded DNA breaks^12,22^. Consequently, our findings of significant elevations in the measured alkaline Comet parameters revealed marked induction of DNA breaks 48 h after exposure of HepG2 cancer cells to Ca(OH)_2_NPs.

The induction of high DNA damage poses a significant threat as it triggers excessive ROS generation and can ultimately result in cell death. Research has shown that even a single DNA break can disrupt the integrity of genomic DNA, leading to cellular death^22–24^. Cellular ROS, highly reactive metabolites, are produced within cells during cellular processes, and play a crucial role in cellular signaling. However, ROS are produced in excess when the balance between oxidants and antioxidants is disrupted; thereby these overproduced ROS attack and cause damage to cellular macromolecules: lipid, proteins, carbohydrates and DNA inducing oxidative stress^25–27^. Excessive ROS generation by Ca(OH)_2_NPs in HepG2 cancer cells was demonstrated by the remarkable elevations noticed in the intensity of fluorescent light emitted from the Ca(OH)_2_NPs-treated HepG2 cells compared to that emitted from untreated HepG2 cells.

As a result, the demonstrated cytotoxicity of Ca(OH)_2_NPs against HepG2 cancer cells in this study may result from the above-mentioned induction of marked DNA breaks and excessive ROS generation in HepG2 cancer cells treated with Ca(OH)_2_NPs because high induction of DNA damage and increased ROS generation forced cells to die through the apoptotic pathway^28,29^. Our data of qRTPCR demonstrated that exposure to Ca(OH)_2_NPs for 48 h trigger apoptosis of HepG2 cells through the concurrent marked upregulation of the apoptotic (p53 and Bax) genes’ expression level and a significant decrease in the anti-apoptotic Bcl2 gene expression level noticed after treatment of HepG2 cancer cells with Ca(OH)_2_NPs.

On the other hand, the non-remarkable changes in of ROS generation level and expression level of p53, Bax and Bcl2 genes noticed after HSF cells treatment with IC50 concentration of Ca(OH)_2_NPs confirmed the safety and non-genotoxicity of Ca(OH)_2_NPs on HSF normal cells. This non-genotoxic effect of Ca(OH)_2_NPs on HSF normal cells may result from the genomic stability and regulation of different DNA repair mechanisms that enable normal HSF cells to maintain genomic DNA and cell integrity^30,31^. However, Ca(OH)_2_NPs cytotoxicity to normal HSF cells can be attributed to the excessive release of calcium ions that directly attack the cell membrane causing loss of its selective permeability and thereby cell death^32^.

The high specificity and increased toxicity of Ca(OH)_2_NPs to HepG2 cancer cells are largely due to the pH differences between normal and cancer cells. Cancer cells are characterized by an acidic extracellular pH (acidic microenvironment), whereas normal cells maintain a more neutral extracellular pH and tightly regulated intracellular pH levels. As a result, the high alkalinity of Ca(OH)_2_NPs induces a more pronounced genotoxic effect on cancer cells due to their acidic environment. Cancer cells are unable to effectively counteract the significant pH changes caused by hydroxide ions and excessive ROS generation, leading to damage to cellular structures, including DNA. In contrast, normal cells, operating in a more neutral pH environment, are better able to manage or resist these pH changes, resulting in less toxicity^33–35^. Consequently, this study demonstrated for the first time the strong cytotoxicity and potent selective genotoxicity of Ca(OH)_2_NPs against cancerous HepG2 cells. Future studies will address the limitations of this study, including the use of only two cell lines, the short exposure time, and the absence of additional molecular analyses, such as flow cytometry and Western blot.

## Conclusion

Collectively from the data discussed above, it was concluded that Ca(OH)_2_NPs induced concentration-dependent cytotoxicity on normal HSF and cancer HepG2 cells. However, exposure to Ca(OH)_2_NPs at IC50 concentration for 48 h was non-genotoxic in normal HSF cells and selectively disrupts the genomic DNA integrity of HepG2 cancer cells through induction of high DNA damage, excessive ROS generation and alterations of the expression level of apoptotic genes. Therefore, further studies on the biological and toxicological properties of Ca(OH)_2_NPs along with possibility of their usage in cancer therapy are recommended.

## Data Availability

The datasets used and/or analyzed during the current study are available from the corresponding author on reasonable request.
